# Book Review

**Published:** 1897-06

**Authors:** 


**A Handbook of Surface Anatomy and Landmarks.** By BERTRAM C. A. WINDLE, M.D. Second Edition. Revised and enlarged in collaboration with T. MANNERS-SMITH. Pp. 143. London: H. K. Lewis. 1896.—In common with all teachers of anatomy, the authors of this really excellent little book seem to have found that it is difficult to get any student of that subject to trouble about the anatomy of the surface. Students in Birmingham are evidently in as much haste to "see inside" as students of other schools. From the fact, however, that a second edition has been called for, it is probable that the authors have to a certain extent remedied this. The book is wonderfully free from mistakes. We would point out a grammatical error on page 14, line 19, and a slip of the pen on page 30, line 21, where the word "coronal" is evidently intended to be "sagittal." In connection with the notice of the ensiform cartilage, the essential importance of taking all measurements from its base should have been emphasised. Several coloured plates have been introduced, and in most cases with advantage; but we scarcely think that figure 11 of the kidney and spleen in their mutual relations and the relations of the former to the vertebral column is accurate—that is, if recent developments be at all correct. The kidneys are much too wide in proportion to their length, and they are certainly too far away from the vertebral column. It would have been well to have given some indication of the position of the kidneys from the front, as most students have but the foggiest notion of it. We think it would have been better had the names of structures to be located been printed in some distinctive type. However, despite these drawbacks, we have no hesitation in strongly recommending the book, and we wish it the success it deserves.

**Hints on Elementary Physiology.** By Florence A. Haig-Brown. Pp. xii., 121. London: J. & A. Churchill. 1896.—This little book is written from notes taken by Miss Haig-Brown from lectures delivered to nurses at St. Thomas's Hospital. It is avowedly written for the help of nurses; but we must frankly confess that we should strongly advise all those who desire some elementary knowledge of physiology to read one of the numerous excellent primers which may be procured, and not to touch this astonishingly incorrect compilation. We are informed that the "heart is situated in the *mediastinum*, or centre of the thorax, in an upper direction from right to left;" that each cavity contains two ounces of blood, and that the function of the chordæ tendineæ is to *open* the valves; that a "*tumour* is a degenerating mass of cells"; that "in the glands of the liver, kidneys and skin the epithelial cells are globular;" that "air exhaled from the lungs will be found to contain . . . no oxygen;" and that one function of the kidney is to "get rid of albuminous matter not used up in digestion." These and other luminous statements concerning the human body, which is truly, according to Miss Haig-Brown, "fearfully and wonderfully made," render the book extremely well fitted to create confusion in the mind of the novice. There are many other errors besides those instanced above, and we are surprised to find that the book is sanctioned and introduced to the public by a physician to a London hospital. The claims of the authoress are, it is true, modest; but ignorance of a subject is no excuse for writing a book on it.

**Section Cutting and Staining.** By W. S. Colman, M.D. Second Edition. Pp. viii., 160. London: H. K. Lewis. 1896.—Dr. Colman's book is a simple and unpretentious one, giving an account of histological methods. Successive chapters are devoted to hardening processes, section-cutting, section-mounting, and staining-methods, and there are in addition special accounts of the most recently devised stains for the central nervous system and for micro-organisms. To those who do not wish such an elaborate work as Lee's, this book may be safely recommended.

**Guide to the Examination in Practical Chemistry for the Conjoint Board.** By Percy A. E. Richards. Pp. 57. London: Baillière, Tindall & Cox. 1896.—This does not profess to be a guide to practical chemistry generally, but follows faithfully the syllabus drawn up by the Examining Board of the Conjoint Colleges. It contains a fairly good description of the tests and processes prescribed, and may be said to fulfil the not very ambitious purpose to which the author has addressed himself.

**Royal Infirmary Cliniques.** By Alexander James, M.D. Pp. 167. Edinburgh: Oliver and Boyd. 1896.—This series of clinical lectures may be of especial interest to the students of the Clinical Medicine Class at the Edinburgh Royal Infirmary, but the clincal lecture method of instruction is too diffuse for those who have to make themselves familiar with the varieties of medical literature beyond the text-book area. The lectures give an interesting account of many cases; but if every hospital physician is going to publish his clinical lectures, even the world itself would not contain the books that should be written.

**The Causes and Treatment of Rheumatoid Arthritis.** By Samuel Hyde, M.D. Pp. xi., 83. London: John Bale & Sons. 1896.—The writer of these few pages sets out by saying that he will pass over briefly the etiology, diagnosis and pathology of the affection, in order to deal thoroughly with the question of treatment. However, it is found that 36 out of his 80 pages are devoted to these topics. The etiology, symptoms, and diagnosis are discussed along the well-known lines, and nothing fresh is brought forward. The chapter on pathology, in which there are some curious and interesting features, is characterised by the writer's wish to adopt all theories; thus he first gives in his adherence to the theory of a neural cause, he then says that rheumatoid arthritis is due to perverted internal secretions, and lastly, the discovery of a specific bacillus being announced, he says that his "own clinical experience has long suggested" such an organism. The writer is all things to all theories, that he may by all means save—himself. There is nothing very noteworthy in the treatment proposed, and we doubt whether anyone who purchases the book will be satisfied with his outlay.

**The Enlarged Cirrhotic Liver.** By Arthur Foxwell, M.D. Pp. 30. Birmingham: Cornish Brothers. [1896].—This essay on the two varieties—the non-alcoholic and the alcoholic—of liver-disease is really an analysis of 202 cases. In only seven out of the total was the liver small; in 90 its size was unchanged, and in 105 it was larger than usual. It appears then that enlargement of the liver is present in more than half the cases of disease of this organ, and that contraction is less characteristic of cirrhosis than is commonly believed, "those cases where diminution is noted being quite the exception."

**Our Baby.** By Mrs. LANGTON HEWER. Fourth Edition. Pp. vii., 140. Bristol: John Wright & Co. 1896.—So long as Mrs. Hewer confines her advice to what we may call the domestic care of the baby she does very well, but when she enlarges on the troubles that the infant flesh is heir to and recommends dosing with aconite, "bromide" and other drugs, we must express an emphatic opinion that not only has she exceeded her duties, but may lead mothers to the very erroneous idea that with the help of this book they can treat their infants' ailments without skilled advice. In the last two chapters—those to which we presume the "London physician," of whom we read in the preface, has given his attention—there are too many instances of a little knowledge being a very dangerous thing for us to quote, but we must enter our strongest protest against the almost indiscriminate use of brandy for infants which Mrs. Hewer advises. It is a drug of inestimable value when called for, but it may be, and often is, grossly abused, and in our opinion should never be given without the sanction of the family doctor—certainly not on the mother's responsibility.

**Notes on the more Common Diseases of the Eye.** By ROBERT W. DOYNE. Pp. vii., 47. London: H. K. Lewis. 1896.—The author in his preface explains that "these few pages" are intended for the practitioner who has by him some good text-book of ophthalmology, but who has not sufficient familiarity with eye diseases to enable him to gain much by reading it. Mr. Doyne's booklet is clearly and forcibly written, the treatment recommended is sound in principle, and some of the suggestions are original, though we cannot endorse the method of uniting the lids by a suture which he advises as a cure for spasmodic entropion. Still, the whole shows that the author has appreciated the difficulty of many a doctor in treating a case of eye disease. But he has not met that difficulty in the least. As an introduction to the study of an ordinary text-book of ophthalmology, it is superfluous. As a guide to diagnosis and treatment, it is inadequate—the less the experience of the practitioner, the more minute should be the details of diagnosis and treatment. To take one example out of many: "Discolorisation (*sic*) of the iris" in iritis is vague, and does not necessarily imply that blue irides become greenish; also, it is useless to mention keratitis punctata without saying how it is to be seen and recognised; and the frequency of application, and not only the strength, of atropine drops should be mentioned. The book in various places seems to presuppose some knowledge of refraction on the part of the practitioner, and the seven pages devoted to that subject contain no guidance for him whatever, and might very well have been omitted. To hint that anyone may safely diagnose and treat tobacco amblyopia without making an ophthalmoscopic examination is, to say the least, injudicious. The style of the book throughout is graphic, and there is very much that is good and to the point; but inasmuch as it is a positive discouragement to the use of the ophthalmoscope, and an attempt to provide a "short cut" to the efficient treatment of eye diseases, we cannot express our approval of it as a whole.

**Short Contributions to Aural Surgery.** By Sir WILLIAM B. DALBY, M.B. Third Edition. Pp. xi., 143. London: J. & A. Churchill. 1896.—These papers were originally published in the *Lancet* and *British Medical Journal*, and have been since collected in book form. Five additional papers now appear in this edition, making nineteen in all. They are marked by the strong common-sense which characterises the author, and being illustrated by numerous cases drawn from his rich experience they form a valuable contribution to the literature of ear disease.

**Voice Production.** By PAUL MAHLENDORFF. Pp. 32. London: P. Dabiero. [N.D.].—If there be any confusion of mind as to methods of voice production before beginning the perusal of this pamphlet, it will be worse confounded should the reader be able to reach the last page.

**The Consumption of Opium in India.** Pp. 51. Calcutta: Office of the *Indian Medical Record*. 1895.—The consumption of opium in India is a subject which has engaged the attention of philanthropists, hygienists, and politicians for many years. There are many excellent and estimable persons who regard its cultivation in India and its sale there and in other countries under the sanction of the Government as a national sin and dishonour. There are others equally worthy of respect who maintain that the moderate use of opium is not only harmless, but in many respects beneficial to the people of India, and that it would be a cruel injustice to prevent its cultivation or to interfere with its sale. A Royal Commission was held in 1893-94 to investigate the whole question. The principal medical member of this Commission was Sir William Roberts. The report which he presented as a member of the Commission was, we may say, entirely favourable to the use of opium. Sir William, it should be remembered, has never been in India, and in this pamphlet it is maintained that this fact should have excluded him from being on the Commission at all. On the other hand, a great deal of the evidence he has collected was from experienced Indian medical officers. Dr. Crombie, of Calcutta, asserts that to deprive the people of Eastern Bengal of opium "would be a terrible and wanton cruelty," for he says it is the only medicine available for their use when malarial fever is prevalent. The *Critique*, which is the subject of these remarks, utterly denies nearly every statement made in Sir William's memorandum. The opponents of habitual use of opium maintain that the health and the morals of the people suffer from the production of opium. They say that wherever opium is grown, it is eaten, and the more it is grown, the more it is eaten, and that opium cultivation originates the habit in rural districts. The writers of this pamphlet have said all that is to be said against Sir William Roberts's memorandum.

**Difficulties under the Infectious Disease (Notification) Act.** Pp. 23. London: The *Lancet* Office. 1895.—This reprint of four articles from the *Lancet* contains the points of difficulty which have occurred in the administration of this Act in England. The remarks and suggestions are in general practical and correct.

**Report of the "Lancet" Special Commission on the Relative Efficiency and Cost of Plumbers' Work.** Pp. 90. London: The Offices of the *Lancet*. 1896.—This booklet, the matter of which appeared in the *Lancet*, should prove of use to architects and others having to deal with estimates and plans for constructional work in house drainage. The work is edited in a practical manner, and the information is generally sound.

**Transactions of the Medical Society of the State of New York.** Published by the Society. 1896.—A special feature of this volume is the report of discussions, in which special aspects of the question were allotted to different speakers. Among these the two most important and interesting are on cerebral and abdominal surgery. Brain tumours were allotted to Dr. Starr, and his paper is a valuable contribution, based as it is on an analysis of 137 cases culled from all sources, and examined with his own special knowledge of the subject. He concludes that only about 7 per cent. of all cases are operable, although relief of intracranial pressure alone may often give rise to considerable improvement, and may, he thinks, aid the absorptive power of drugs. Focal epilepsy forms another division of the discussion. Starr (who also touched on this) expressed himself as having been disappointed with the after-results of operation, the attacks recurring, whether they were spontaneous or traumatic in origin. Sachs thinks trephining justifiable only in the earliest periods after a traumatic injury before secondary degeneration is established. Among the surgical speakers the bone-flap operation was most favoured. Nothing new or very hopeful could be said for craniotomy for idiocy and imbecility. In the discussion on abdominal surgery Vander Veer reported three cases of intestinal anastomosis by means of Murphy's button, and discussed this and other methods. The dangers of the button were admitted, but he thought that at present it was the best method for circular enterorrhaphy in the small intestine, or for anastomosis between the latter and the stomach or gall-bladder, but not in the case of the large gut where solid fæces are apt to plug the lumen. The rather startling statement is quoted from Levings, of Milwaukee, that Cushing's or Connel's suture, for end-to-end union, are both speedier methods than that by Murphy's button. For union between large and small intestine, or in resection of the large gut, the author thinks Maunsell's method will prove the best. Cæsarian section and vaginal hysterectomy are also included in the discussion. Among many isolated papers, of varying interest, one of the most interesting (if not quite convincing) is that by Dr. Willy Meyer on the early diagnosis of tuberculosis of the kidney. He affirms the lesion to be always primarily unilateral, reaching the other kidney by passing down the ureter to the bladder and thence up the opposite one. When the caseating matter in the kidney softens and reaches the pelvis of the kidney, it passes on to the bladder, inoculating it around the ureteral opening, and infecting the ureter on the way. If at this stage cystoscopy be practised, Dr. Meyer asserts that by injection around this opening, and "a number of circumscribed, clearly defined, inflamed areas of the mucous membrane . . . . between it and the slightly hyperæmic trigonum," vesical tuberculosis can be recognised in its beginnings. Bacteriological staining of the deposit in the urine will demonstrate the bacillus; and finally Dr. Meyer is convinced that he can (by Kelly's method in the female, and the use of Casper's ureter-cystoscope in the male) collect the urine from each ureter separately, under the direct guidance of the eye. This were, indeed, "a consummation devoutly to be wished." Supposing the diagnosis established, extirpation of the kidney, if done early enough, will result in a cure. Dr. Halsted Myers has a good paper on congenital dislocation of the hip, in which he reviews the various methods of treatment advocated. These transactions form a well-bound and excellently printed volume, and contain (besides matter relating to the routine of the Society) communications ranging over a wide area of medical practice.

**Transactions of the Michigan State Medical Society.** Vol. XX. Grand Rapids: Published by the Society. 1896.—We have here a substantial volume of over 800 pages, containing the President's address, the annual addresses on Medicine, Surgery and Gynæcology, the reports of several papers read in the Surgical Section and many in that of Medicine and Obstetrics, and the discussions which followed the reading of these papers. Dr. Carl Huber's paper upon nerve suturing and nerve implantation is a record of careful experimental and observational work. He shows that the best method of operating for loss of substance in a nerve trunk is that of implanting a segment taken from another nerve. Better results are yielded by this method than by the others which have been adopted. Dr. C. B. Nancrede gives details of two large and rapidly-growing tumours of nerve trunks of benign nature which he was able to remove without destroying the continuity of nerves affected. There are three papers on typhoid fever: on the pathology by Dr. Henry B. Baker, on the symptoms by Dr. Hugh McColl, and on the treatment by Dr. Duffield. The advantages of the cold bath treatment in this disease were well brought out in the subsequent discussion. The volume contains many other instructive and interesting papers, and fully deserves an index.

**Transactions of the Clinical Society of London.** Vol. XXIX. London: Longmans, Green, and Co. 1896.—This volume well maintains the reputation of the Society in presenting a number of clinical cases well worthy of record. Without particularising individual papers, we may say that the cases are uniformly described with great care and thoroughness, so that the reader is enabled to realise the important points of their clinical aspect from the descriptions given. The volume will repay a careful study.

**Proceedings of the West London Medico-Chirurgical Society.** Vol. VII. London: John Bale & Sons. 1896.—As the Society which is responsible for this volume now issues a quarterly journal, we thought we had seen the last of these belated volumes. This one, bearing on its preface the date of September 30th, 1896, begins with a presidential address on October 5th, 1894, and continues with a report of the other meetings held in 1894–95. As if this were not bad enough, the editors have misprinted the adjourned meeting in November of 1894 as 1893. Sir James Crichton Browne's lecture on "Dreamy Mental States," which occupies more than a fifth of the volume, was issued separately long ago, and was reviewed by us in June, 1896. Although that communication is only one of many interesting papers, the West London Medico-Chirurgical Society might have spent its money in a better way than in printing this book.

**Transactions of the American Association of Obstetricians and Gynecologists.** Vol. VIII. Philadelphia: Wm. J. Dornan. 1896.—Although it is impossible to notice all the excellent papers in this volume, special attention must be directed to one on the treatment of puerperal sepsis by Dr. A. B. Miller, who advocates an entirely new treatment. He says: "In consequence of the degeneration of the human race and the teaching of the obstetrical art, women are placed in the recumbent posture during confinement and during their convalescence. Owing to the anatomy of the birth-canal only an imperfect drainage can be secured in this posture. The lochia from the womb form a pool in the vault of the vagina, and the cervical neck is constantly bathed by resting in the accumulation. So here we have the broth or suitable culture-medium and the warmth of the body to develop the micro-organisms of puerperal sepsis—an incubator the envy of our most modern bacteriologists." Therefore the treatment should not be continuous syringing, but dry-packing the vagina with gauze or any suitable material, and changing when necessary to keep the whole tract absolutely dry. This is certainly worth a trial. A paper by Dr. Marcus Rosenwasser on ruptured ectopic pregnancy emphasises what we already know, viz., the absolute necessity of immediate operation when the diagnosis is made. Dr. T. J. Maxwell describes some very interesting anomalies found in abdominal surgery, thereby warning the neophyte to be prepared for any emergency, or avoid the sacred precincts of the peritoneum which have now become "the fools' paradise." We should have liked to have seen the papers better illustrated. The "In Memoriam" portraits are well done. Among them, with an obituary notice, is one of Dr. Boisliniere, whose latest book we have reviewed in another part of this number.

**Transactions of the Dermatological Society of Great Britain and Ireland.** Vol. II. London: H. K. Lewis. 1897.—In this second report we have about seventeen pages less than in the first one, which should hardly be the case in a growing society; but we must admit there has been some very good work done, and quantity is not everything. The President, Dr. Pye-Smith, in the opening address, claimed for dermatology that it should be considered as a part only of general medicine, and that, therefore, its pathology should be based on the broad and ever-flowing stream of general pathology. Without advocating the absurdities of hydropathy, he claimed benefit from a liberal supply of water, and weak vegetable infusions, as an important part of general dieting in the treatment of various skin affections, and by no means withheld the use of light wines, porter or beer, in chronic dermatitis. We can endorse these opinions, and also the statement that "what most people eat is for most people wholesome, and what a natural appetite finds appetizing seldom disagrees." Brandy in small quantities, in young children with general eczema, is often very salutary. He concluded a very sensible and practical address with a quotation (which we deem very wise) from Hebra, viz.: "He will obtain the best success in treatment who is not always changing his methods, but persists in the use of whatever plan is indicated with patience and determination." Dr. H. Radcliffe Crocker read a paper on the internal treatment of psoriasis, and some other skin affections, with salicylate of soda. He produced good evidence of its efficacy, and his views were well supported by many of the members. A conspicuous feature of the society's work was the exhibition of a large number of patients at the general meetings.

**The American Year-Book of Medicine and Surgery.** London: The Rebman Publishing Co. (Ltd.). Philadelphia: W. B. Saunders. 1897.—This, the second number of Gould's year-book, is a handsome volume of 1257 pages, profusely illustrated, and much improved by a new and useful feature, each article being preceded by a brief recapitulation of the "trend and results of the year's work." We cordially endorse the editor's view "that it is precisely this feature that will make the book still more acceptable to those whom the demands of practice leave little time to settle matters of controversy and doubt, and who must therefore welcome the ripe decisions of those most capable of pronouncing them." Among other evidences of progress in general medicine, Drs. Pepper and Stengel call attention to the discovery of the specific cause of the bubonic plague by Kitasato, and the method of Elsner for the diagnosis of typhoid fever, and they remark that "nothing is more refreshing in the study of a year's medical work than the evidence exhibited in the literature of our own countrymen, as well as in that of foreign nations, that experiment and investigation are taking the place of speculation." They further remark that "nothing of moment has been added to the knowledge of the bacillus of typhoid fever or its life-history," and further, "unfortunately, the bacteriologist has hitherto given little aid, and the chemical examinations of the urine and other clinical methods have been unavailing in doubtful cases." Now it is just in those doubtful cases that we are able to derive assistance from the diazo reaction of Ehrlich, and from the bacteriological test of Widal, both of which have given positive results in cases of typhoid, whereas in meningitis the results have with both tests been negative. By some curious oversight those tests about which much has been written during the past year do not appear to have been considered worthy of even a passing mention. We are sorry to be obliged to endorse the remark that "the attempts to apply the methods of serum therapeutics to this disease have thus far met with little encouragement," and we heartily agree in the criticism of Osler that "a greater minuteness of attention to diagnosis might frequently be desirable in the reports of brilliant results." As regards tuberculosis, the authors remark that "little has been added to previous knowledge of this disease by the contributions of the past year. . . . Serum-therapy has been lauded. . . . The evidence, however, justifies no deductions. More laboratory and less clinical work in this direction are desirable." The "antiphthisin" of Klebs, which appears to be a highly diluted tuberculin, has given excellent results in the hands of Von Ruck, but the editors suggest that perhaps the climate of Asheville may be partially responsible for the success. In an extensive work like this it is impossible to avoid omissions, but nevertheless it is remarkable that, under the heading of Rheumatoid Arthritis, there should be no mention of the discovery of a bacillus which Drs. Bannatyne, Blaxall, and Wohlmann believe to be the true cause of the disease. (See *Lancet*, April 25th, 1896.) We must refrain from further comment, and conclude by remarking that Dr. G. Gould and his staff of coadjutors appear to have done their rather difficult task of choosing and condensation with great skill, and that the editorial comments are for the most part very judicious and useful.

**The Journal of Experimental Medicine.** New York: D. Appleton and Company.—We are glad to welcome this very valuable addition to our Exchange-list, and to learn that the first year of its existence has been one of gratifying prosperity. It is devoted to the publication of original contributions to the medical sciences from American sources. The expense of publishing it in its present form must be considerable, and we trust that the *Journal* will meet with that extended support which its editor, Dr. W. H. Welch, claims for it, and which it certainly deserves. The first volume contained a great many papers of originality and value, and the opening number of the second volume gives rich promise that the exceptionally high character of this *Journal* will be maintained. This number contains a paper by Dr. H. H. Young on the presence of nerves in tumours as revealed by a modification of Ehrlich's method of staining *intra vitam* with methylene blue. Messrs. G. B. Magrath and H. Kennedy have a contribution on "The Relation of the Volume of the Coronary Circulation to the Frequency and Force of the Ventricular Contraction in the Isolated Heart of the Cat," in which they show that the force of the ventricular contraction varies directly with the change in the amount of blood supplied to the cardiac muscle, whilst the frequency shows no such relation. They further found that distension of the left auricle and ventricle lessened the flow through the coronaries, and that a heart from which the blood-supply had been almost entirely cut off showed "staircase" beats, which gave place to normal contractions as the circulation through the cardiac muscle was restored. An account of an elaborate investigation into the time of reflex winking is given by Mr. David P. Mayhew. He found that in 450 experiments on sixteen subjects the mean total time was .042 sec. Natural winks occurring shortly before the record was taken had no influence. Apprehension seemed to shorten the time. Dr. F. Pfaff and Mr. Alfred Balch contribute a full report of analyses of human bile, and the effect of some drugs on its secretion in a case of biliary fistula following cholecystotomy. They are inclined to attach value to the administration of dried ox bile as a cholagogue, as well as in certain cases of constipation, and possibly in cases in which it is desired to increase the absorption of fat. They direct that .25 gramme of dried ox bile should be made into a pill, and from six to fifteen pills given in the day. Articles on "The Influence of Phloridzin on the Bile and Lymph" by Dr. P. A. Levene, and on "The Gelatin from White Fibrous Connective Tissue" by Mr. Van Name, make up an interesting number. The excellent type, good illustrations, and, we may add, freedom from advertisements, make the *Journal* pleasant to read.

**Treatment.** London: The Rebman Publishing Co., Ltd.—We are not much inclined to think that the medical world stands in need of a periodical such as this, the first number of which was issued in March. Each number consists of 24 pages, and the journal is to appear twice a month. The original articles are short and practical, but the abstracts are too much of the "scissors and paste" type. The shabby appearance of the journal is altogether unworthy of the house associated with the magnificent "Blue Cover" Series in which so many excellent volumes have been issued recently.

**La Revue Philanthropique.** No. 1. 10 Mai, 1897. Paris: Masson et Cie.—We have to welcome this monthly magazine, which, under the editorship of M. Paul Strauss, is destined to give many a lesson in universal philanthropy. It opens with an editorial programme, truly described as vast, and embracing a widely extended range of topics. A chapter on Fraternity deals with the tragic occurrence of the Charity Bazaar of the Rue Jean-Goujon, and another chapter on Philanthropy informs us that our Diamond Jubilee is to be distinguished "par la fondation d'un hôpital qui portera le nom du Prince of Wales" (!). A chapter on the feeding of infants advocates the habitual use of sterilised milk. It appears to us that this review is likely to lead a very useful life, and we trust that it may be a prosperous and protracted one.

**Clinical Excerpts.** Elberfeld: Friedr. Bayer & Co.—It was a happy idea of this well-known firm to send out monthly, beginning in January this year, a collection of reprinted articles dealing with the preparations issued by them. When these articles are independent they form a valuable guide to many of the new drugs by which the practitioner is so often tempted. A neat reading-case is supplied in which all the numbers can be kept for reference. This enterprising firm will doubtless be quite ready to send the numbers to anyone asking for them.

